# Diagnostic and prognostic value of navigated transcranial magnetic stimulation to assess motor function in patients with acute traumatic spinal cord injury

**DOI:** 10.1016/j.bas.2025.104229

**Published:** 2025-03-07

**Authors:** Maximilian Schwendner, Marianne Kanaris, Anthony M. DiGiorgio, Michael C. Huang, Geoffrey T. Manley, Phiroz E. Tarapore

**Affiliations:** a Department of Neurosurgery, Heidelberg University Hospital, Ruprecht-Karls-University Heidelberg, Germany; b Department of Neurological Surgery, Zuckerberg San Francisco General Hospital, University of California San Francisco, USA

**Keywords:** navigated transcranial magnetic stimulation, Spinal cord injury, Motor-evoked potentials, Intensive care

## Abstract

**Introduction:**

Traumatic spinal cord injuries (SCI) represent a profoundly life-altering diagnosis.

**Research question:**

The aim of this study was to evaluate the diagnostic and prognostic value of navigated transcranial magnetic stimulation (nTMS) in assessing motor function in the management of patients with acute SCI.

**Material and methods:**

nTMS motor mapping of both upper extremities (UE) and lower extremities (LE) was performed in patients suffering from acute traumatic SCI. Data from intraoperative neuromonitoring (IONM) and patient outcomes, including American Spinal Injury Association Impairment Scale (AISA) scores, were analyzed.

**Results:**

The patients had a mean age of 68.9 ± 15.6 years (range: 28–94 years). Preoperatively, 7 patients (35.0%) were classified as AISA A or B, and 13 (65.0%) were classified as AISA C or D. At follow-up, 5 patients (25.0%) had improved.

In all patients motor evoked potentials (MEPs) were elicited. MEPs of UE muscles were observed in 38 (61.3%) muscles in IONM and 41 (66.1%) muscles in TMS. MEPs of LE muscles were observed in 5 (19.2%) and 7 (26.9%) muscles, respectively. e

Combining the results of IONM and nTMS, a sensitivity of 0.852 and a specificity of 0.889 for motor function of the tested muscle at follow-up was achieved for upper extremity muscles. For lower extremity muscles, a sensitivity of 0.571 and a specificity of 1.00 was achieved.

**Discussion and conclusion:**

nTMS in patients with acute SCI provides an objective assessment of motor system integrity. Despite a relatively low sensitivity, potentially due to decreased excitability, this technique exhibited excellent specificity in predicting short-term and long-term motor outcomes.

## Highlights

• Navigated transcranial magnetic stimulation (nTMS) serves as a non-invasive and precise tool to assess motor function in patients with acute traumatic spinal cord injury (SCI).
• NTMS elicited motor evoked potentials (MEPs) with a similar rate compared to intraoperative neuromonitoring (IONM) and offers an effective alternative for predicting long-term motor outcomes, even outside the operating room.
• Positive MEPs recorded by nTMS strongly correlated with superior American Spinal Injury Association Impairment Scale (AIS) scores at follow-up, emphasizing the potential of nTMS for early prognosis of neurological recovery in SCI patients.

## Abbreviations

ASIA: American spinal injury association impairment scale
APB: Abductor pollicis brevis muscle
BCS: Biceps brachii muscle
BMRC: British medical research council
CT: Computed tomography
DICOM: Digital imaging and communications in medicine
EMG: Electromyography
FU: Follow-up
ICU: Intensive care unit
IONM: Intraoperative neuromonitoring
LE: Lower extremities
MEPs: Motor evoked potentials
MEPs_IONM_: Motor evoked potentials from intraoperative neuromonitoring
MEPs_TMS_: Motor evoked potentials from transcranial magnetic stimulation
MRI: Magnetic resonance imaging
nTMS: Navigated transcranial magnetic stimulation
rMT: Resting motor threshold
SCI: Spinal cord injury
TA: Tibialis anterior muscle
UE: Upper extremities

## 1 Introduction

Spinal cord injury (SCI) is a devastating condition, leading to significant and often permanent changes in the lives of affected patients. Acute SCI typically results from trauma, such as high-velocity motor vehicle accidents, falls, or sports injuries and can lead to significant motor, sensory, and autonomic dysfunction, often resulting in paralysis, loss of sensation, and disruption of autonomic and bowel/bladder control (Kumar et al., 2018; Quadri et al., 2020). The impact of SCI extends beyond the physical domain, affecting psychological well-being and social integration (Kumar et al., 2018; Quadri et al., 2020).

An analysis of survey data from the United States Nationwide Inpatient Sample databases for the years 1993–2012 revealed an annual incidence of traumatic spinal cord injury of approximately 54 cases per million people in the United States, translating to about 18,000 new cases each year (Jain et al., 2015). Over this period, the total number of cases increased, primarily driven by an increased incidence among older adults (Jain et al., 2015). This increase is largely attributed to a significant increase in fall-related injuries (Jain et al., 2015). The associated socioeconomic burden of SCI is substantial, with lifetime costs for care and support of several million United States dollars per individual, depending on the level and severity of the injury and the age of the patient (Malekzadeh et al., 2022; DeVivo et al., 2011). These costs account not only for direct medical expenses and rehabilitation but include assistive devices, home modifications, and long-term care.

Given the heterogeneity of SCI in terms of both injury mechanisms and individual patient responses, there is a critical need for objective, quantifiable methods to assess the current status of patients, predict outcomes, guide clinical decision-making, tailor rehabilitation strategies, and monitor recovery progress. A structured clinical examination alone often falls short in this regard, especially when patients are unable or unwilling to participate in the examination, or when there is variability over time and practitioners. Motor evoked potentials (MEPs) offer a quantifiable and objective measure of the integrity of the motor system with high inter-session consistency. Therefore, the intraoperative application of neuromonitoring, including somatosensory potentials and MEPs has become widespread in surgeries for traumatic spine fractures and SCI ^6,7 8^. It has also been shown to predict neurological outcomes (Dhall et al., 2018). The presence of intraoperative MEPs significantly predicts the American Spinal Injury Association Impairment Scale (ASIA) score and correlates with improvements in motor outcomes for patients with severe SCI (Dhall et al., 2018). For patients outside the operating room, navigated transcranial magnetic stimulation (nTMS) is a well-tolerated and non-invasive method for accurately evaluating MEPs.

The aim of this study was to examine the diagnostic and prognostic value of nTMS to assess motor function in patients with acute traumatic spinal cord injury.

## 2 Methods

### 2.1 Hypothesis

Our hypothesis posits that navigated transcranial magnetic stimulation (nTMS) is a valuable adjunctive tool for assessing motor function in patients with acute traumatic spinal cord injury and predicting their long-term clinical outcomes.

### 2.2 Ethics

This study was approved by the University of San Francisco California Institutional Review Board (16–20684, September 1, 2020). All research was conducted according to the Declaration of Helsinki. Written informed consent was obtained from patients or their appointed decision-maker.

### 2.3 Study protocol

Patients presenting with acute spinal cord injury between January 2022 and July 2024 were considered eligible for this study and were prospectively included. Inclusion criteria required an acute onset of symptoms following trauma, occurring less than 24 h before presentation, cranial computed tomography (CT) imaging that ruled out concomitant brain injuries, absence of contraindications for nTMS, and written informed consent. Exclusion criteria were age below 18 years, general nTMS exclusion criteria such as the presence of cochlear implants or cardiac pacemakers, acute isolation due to infectious disease, acute instability of vital parameters, and patients receiving end-of-life care.

Motor function was assessed and graded according to the British Medical Research Council (BMRC) scale. In addition, the spinal cord injury was graded according to the American Spinal Injury Association (ASIA) classification. These evaluations were conducted preoperatively, 72 h postoperatively (or 72 h post-admission for non-surgical cases), at discharge, and the follow-up visits.

Data from intraoperative neuromonitoring (IONM) using transcranial electric stimulation to monitor MEPs was also considered in the interpretation of the results. IONM was performed during the initial surgery following the spinal cord injury (SCI), which was typically conducted within 24 h of admission. All surgeries were performed under general anesthesia, specifically using total intravenous anesthesia. Muscle relaxants were administered only during intubation and were not continued during the surgery to ensure optimal conditions for neuromonitoring.

### 2.4 nTMS mapping procedure at the intensive care unit

**Fig. 1:**
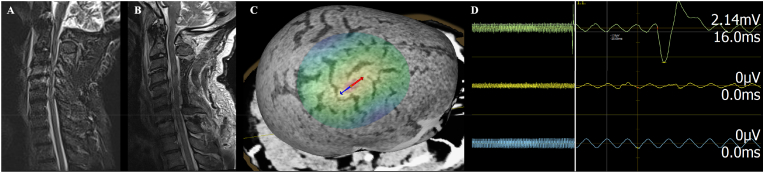
Illustrative case.

Cranial computed tomography (CT) imaging for this study was performed as part of the routine clinical trauma work-up using a Somatom 64-slice CT scanner (Siemens AG, Erlangen, Germany). Although MR imaging is routinely used for neuronavigation, it was not feasible to perform this additional study in an expedient fashion. The CT scans, saved in DICOM (Digital imaging and communications in medicine) format, underwent preprocessing for integration with the nTMS system. Preprocessing included removing non-patient structures, such as the headrest, and optimizing windowing settings to enhance the visibility of cortical anatomy (Schramm et al., 2020). These steps were carried out using the Aliza Medical Imaging and DICOM Viewer (Aliza 1.98.12, Copyright, 2014–2020 Aliza Medical Imaging, Bonn, Germany) (Schramm et al., 2020). The preprocessed CT scans were then imported into the nTMS system (eXimia NBS 5.0; Nexstim Plc., Helsinki, Finland) which is equipped with an infrared tracking system (Polaris Spectra; Polaris, Waterloo, Ontario, Canada), enabling precise neuronavigation during mapping (Fig. 1) (Ruohonen and Karhu, 2010). A head tracker with reflective sphere markers was attached to the patient's forehead to facilitate accurate tracking.

Motor mapping was performed according to clinical routine and current guidelines (Schramm et al., 2020; Krieg et al., 2017). Motor evoked potentials elicited by nTMS (MEPs_TMS_) were recorded using continuous electromyography (EMG) recording, using a six-channel EMG system integrated into the nTMS device that records both the stimulation and the resulting EMG responses in real time (Schramm et al., 2020). For EMG recording, pre-gelled surface electrodes (Neuroline 700; Ambu, Ballerup, Denmark) were applied to record the activity of the biceps brachii muscle (BCS) and abductor pollicis brevis muscle (APB) for the upper extremity and tibialis anterior muscle (TA) for lower extremity (Fig. 1) (Schramm et al., 2020). The muscles recorded are standard choices for nTMS motor mapping as they consistently yield reliable results based on clinical experience. Additionally, BCS and APB represent the upper and lower cervical nerve roots of the cervical spine (BCS: C5/6; APB: C8/Th1). Special attention was given to optimizing EMG recording and minimizing noise by determining optimal electrode placement, including the grounding electrode. MEPs_TMS_ were considered valid based on amplitude (above 50 μV, with higher amplitude thresholds in the presence of unavoidable EMG noise), latency (10–35 ms for upper extremity musculature, 30–60 ms for lower limb musculature), and signal morphology (Krieg et al., 2017). Initial rough mapping was conducted to identify potential motor hotspots. If no MEPs were elicited, stimulation intensity was incrementally increased until either MEPs were detected, the patient reported discomfort, or further increases in stimulation intensity were limited by the stimulator output. Following this, the patient's resting motor threshold (rMT) was determined. The complete extent of the MEP-positive stimulation sites was then examined at 105% of the rMT. For mapping of TA muscle, mapping was performed starting with an intensity of 130% rMT.

After the nTMS session, EMG recordings from all stimulation sites were reviewed using analysis software (eXimia NBS 5.0; Nexstim Plc., Helsinki, Finland) to identify and rule out false-positive and false-negative errors.

### 2.5 Data analysis

Statistical analyses were conducted using Prism (version 8.4.1; GraphPad Software, La Jolla, CA, USA). Descriptive statistics, including mean, median, minimum, maximum, and standard deviation, were calculated for patient-related characteristics and clinical examination data. Additionally, clinical findings were compared with nTMS and IONM results to evaluate their correlation and potential predictive value. A level of significance was set at p < 0.05 for all statistical analyses.

## 3 Results

### 3.1 Patient characteristics

This study included 20 patients with acute spinal cord injury, aged 68.9 ± 15.6 years (28–94 years). The average length of hospital stay was 31.4 ± 31.3 days (6–105 days) (Table 1). Maximum follow-up data was available 162 ± 161 days (13–545 days) after the initial trauma (Table 1).

**Table 1:** Neurological status.

		Time-point	preOP	postOP/72h	TMS	Discharge	Follow-Up
		**Time after surgery** (days; Mean ± SD(Min-Max))			4.3 ± 4.3 (0–14)	31.4 ± 31.3 (6–105)	162.2 ± 160.7 (13–545)
**ASIA Impairment Scale** (n(%))							
		A	4 (20.0%)	4 (20.0%)	4 (20.0%)	3 (15.0%)	3 (15.0%)
		B	3 (15.0%)	2 (10.0%)	2 (10.0%)	1 (5.0%)	1 (5.0%)
		C	4 (20.0%)	5 (25.0%)	3 (15.0%)	5 (25.0%)	4 (20.0%)
		D	9 (45.0%)	9 (45.0%)	11 (55.0%)	11 (55.0%)	12 (60.0%)

Table 1 illustrates the neurological status per the American Spinal Injury Association (ASIA) Impairment Scale. Examinations were performed preoperatively (preOP), 72 h postoperatively (postOP) or 72 h post-injury (72h) in case of non-surgical treatment, as well as at discharge and on last follow-up.

### 3.2 Clinical data

7 patients (35.0%) were classified as ASIA A or B, while 13 patients (65.0%) were classified as ASIA C or D on initial examination at admission. The level of injury was located in the cervical spine in 15 cases (75.0%) and the thoracic spine in 5 cases (25.0%) (Table 2). Upon postoperative examination, one patient improved from ASIA B to C. Additionally, 4 patients (20.0%) showed improvement by discharge, including 2 patients who improved by two scale levels (from ASIA A to C and ASIA B to D). At follow-up 162.2 ± 160.7 days post-injury, 5 patients (25.0%) had improved, including 2 patients improving by two levels (from ASIA A to C and ASIA B to D). No cases of deterioration in ASIA scale levels were observed. Detailed data is further illustrated in Table 1.

**Table 2:** Level of injury.

Level of injury	ASIA Impairment Scale			
	A	B	C	D
**C2** n (%)	–	–	–	1 (5.0%)
**C3** n (%)	–	–	–	1 (5.0%)
**C4** n (%)	–	–	3 (15.0%)	2 (10.0%)
**C5** n (%)	2 (10.0%)	–	–	3 (15.0%)
**C6** n (%)	–	1 (5.0%)	–	–
**C7** n (%)	–	1 (5.0%)	–	1 (5.0%)
**Th10** n (%)	1 (5.0%)	1 (5.0%)	–	–
**Th11** n (%)	–	–	–	1 (5.0%)
**Th12** n (%)	1 (5.0%)	–	1 (5.0%)	–

Table 2 shows the level of injury and corresponding initial examination according to the American Spinal Injury Association (ASIA) Impairment Scale for all 20 patients included in this study.

### 3.3 nTMS examination

The nTMS motor mapping was successfully performed 4.5 ± 4.1 (1–14) days after trauma in all cases. In 5 cases (25.0%) with cervical SCI, no mapping of LE was performed due to the complete absence of neurological impairments to the LE. In 100% of patients and 87.5% of hemispheres, MEPs_TMS_ were elicited, with a minimum resting motor threshold of 68% ± 20% (37%–100%) of the maximum stimulator output. In total, MEPs_TMS_ recordings of 80 UE muscles were available, with nTMS eliciting MEPs_TMS_ in 47 muscles (58.8%) (Table 3). For LE MEP recording of 30 muscles was performed, with MEPs_TMS_ recorded in 9 (30.0%) (Table 3). MEP latencies were 14 ± 1 (12–17) msec for BCS, 26 ± 6 (20–37) msec for APB, and 43 ± 5 (35–49) msec for TA. For clinical motor activity in the corresponding muscle, positive predictive values for UE were 97.9% at the time point of examination, 100% at follow-up, and for LE 100% at the time point of examination and follow-up. As motor status improved, comparing the time point of the nTMS examination to follow-up (FU), the corresponding negative predictive values and sensitivity decreased (Table 4). Furthermore, a ratio of positive MEPs_TMS_ for the corresponding muscles below the level of injury was calculated and correlated with the ASIA classification at FU. When no MEPs_TMS_ were elicited, no motor function at FU was observed (ASIA A + B). For a ratio of positive MEPs_TMS_ ≥ 0.66, all patients showed ASIA D (Table 5). If any MEPs_TMS_ of the M. tibialis anterior could be elicited, the neurological status at FU was ASIA D in this cohort.

**Table 3:** Correlation of muscle strength and motor-evoked potentials in the navigated transcranial magnetic stimulation examination.

	UE at nTMS	LE at nTMS	UE at Follow-Up	LE at Follow-Up
**Motor status n(%)**				
- BMRC 0/5 - BMRC 1–3/5 - BMRC 4/5 - BMRC 5/5	14 (17.5)	16 (53.3)	9 (11.3)	9 (30.0)
	22 (27.5)	3 (10.0)	18 (22.5)	5 (16.7)
	14 (17.5)	4 (13.3)	20 (25.0)	8 (26.7)
	30 (37.5)	7 (23.3)	33 (41.3)	8 (26.7)
**Overall MEP findings n(%)**				
- Positive - Negative	47 (58.8)	9 (0.30)	n.a.	n.a.
	33 (41.3)	21 (0.70)		
**Ratio of positive MEPs**				
- BMRC 0/5 - BMRC 1–3/5 - BMRC 4/5 - BMRC 5/5	0.07	0.00	0.00	0.00
	0.59	0.33	0.44	0.00
	0.79	1.00	0.70	0.50
	0.73	0.57	0.76	0.63

Table 3 illustrates the distribution of muscle strength grades according to the British Medical Research Council Scale (BMRC) and the distribution of positive and negative motor-evoked potentials (MEPs) for upper extremity (UE) and lower extremity (LE) muscles. For UE, the M. biceps brachialis and M. abductor pollicis brevis were examined. For LE, the M. tibialis anterior was selected. In addition, the ratio of positive MEPs ($\frac{number\;of\;positive\;MEPs}{number\;of\;all\;MEPs}\;)$ was calculated for each BMRC grade. Analysis were performed for the time point of the navigated transcranial magnetic stimulation (nTMS) examination and at follow-up.

**Table 4:** Correlation between the clinical motor status and the findings in intraoperative neuromonitoring and navigated transcranial magnetic stimulation.

	Positive predictive value	Negative predictive value	Sensitivity	Specificity
**Positive MEPs in IONM**				
- UE BMRC ≥1 at 72h - UE BMRC ≥1 at PRE - UE BMRC ≥1 at FU - LE BMRC ≥1 at 72h - LE BMRC ≥1 at PRE - LE BMRC ≥1 at FU	0.756	0.755	0.820	0.678
	0.833	0.736	0.823	0.750
	0.885	0.434	0.697	0.719
	0.743	0.707	0.605	0.820
	0.743	0.707	0.605	0.820
	0.943	0.431	0.500	0.926
**Positive MEPs in nTMS**				
- UE BMRC ≥1 at nTMS - UE BMRC ≥1 at FU - LE BMRC ≥1 at nTMS - LE BMRC ≥1 at FU	0.979	0.394	0.697	0.929
	1.00	0.273	0.662	1.00
	1.00	0.762	0.643	1.00
	1.00	0.429	0.429	1.00
**Positive IONM and/or nTMS**				
- UE BMRC ≥1 at FU - UE BMRC ≥4 at FU - LE BMRC ≥1 at FU - LE BMRC ≥4 at FU	0.979	0.500	0.852	0.889
	0.808	0.875	0.950	0.609
	1.00	0.500	0.571	1.00
	0.917	0.667	0.647	0.923

In Table 4, the correlation between positive motor evoked potentials during intraoperative neuromonitoring (IONM) and navigated transcranial magnetic stimulation (nTMS) with muscle activity according to the British Medical Research Council (BMRC) motor function score is presented. This correlation was assessed for motor function preoperatively (PRE), 72 h post-injury (72h), at the time of the navigated transcranial magnetic stimulation examination (nTMS), and at follow-up (FU). The analysis includes data for upper extremity (UE) and lower extremity (LE) muscles.

**Table 5:** Correlation between the American Spinal Injury Association (ASIA) Impairment Scale and motor evoked potentials.

ASIA Impairment Scale	A	B	C	D
**Cervical SCI nTMS**				
- Number of patients - Ratio of positive MEPs (Mean ± SD(Min-Max))	2	1	2	5
	0.00 ± 0.00 (0.00–0.00)	0.00 ± 0.00 (0.00–0.00)	0.33 ± 0.00 (0.33–0.33)	0.47 ± 0.18 (0.17–0.67)
**Cervical SCI with nTMS only for UE**				
- Number of patients - Ratio of positive MEPs (Mean ± SD(Min-Max))	0	0	0	5
				0.75 ± 0.25 (0.50–1.00)
	
**Thoracic SCI nTMS**				
- Number of patients - Ratio of positive MEPs (Mean ± SD(Min-Max)	1	0	2	2
	0.00 ± 0.00 (0.00–0.00)		0.00 ± 0.00 (0.00–0.00)	0.50 ± 0.00 (0.50–0.50)
**Cervical SCI nTMS + IONM**				
- Number of patients - Ratio of positive MEPs (Mean ± SD(Min-Max))	2	1	2	5
	0.00 ± 0.00 (0.00–0.00)	0.00 ± 0.00 (0.00–0.00)	0.33 ± 0.00 (0.33–0.33)	0.70 ± 0.22 (0.50–1.00)
**Cervical SCI with nTMS + IONM only for UE**				
- Number of patients - Ratio of positive MEPs (Mean ± SD(Min-Max))	0	0	0	3
				0.92 ± 0.14 (0.75–1.00)
**Thoracic SCI nTMS + IONM**				
- Number of patients - Ratio of positive MEPs (Mean ± SD(Min-Max)	1	0	2	1
	0.00 ± 0.00 (0.00–0.00)		0.25 ± 0.35 (0.00–0.50)	1.00 ± 0.00 (1.00–1.00)

Table 5 correlates the American Spinal Injury Association (ASIA) Impairment Scale ratings for spinal cord injuries (SCI). The ratio of muscles below the level of injury showing positive motor evoked potentials (MEPs) during intraoperative neuromonitoring (IONM) and navigated transcranial magnetic stimulation (nTMS) motor mapping. In 5 patients with fully intact motor function of the lower extremities, nTMS was only performed for the upper extremity (UE).

### 3.4 IONM data

Corresponding IONM data was available in 17 (85.0%) cases. For clinical motor activity in the corresponding muscle, positive predictive values for UE were 83.3% preoperatively and 88.5% at follow-up, 74.3% preoperatively, and 94.3% at follow-up for LE. As motor status improved from the time point of the nTMS examination to follow-up, the corresponding negative predictive values and sensitivity decreased (Table 4).

### 3.5 MEPs in IONM and nTMS

**Fig. 2:**
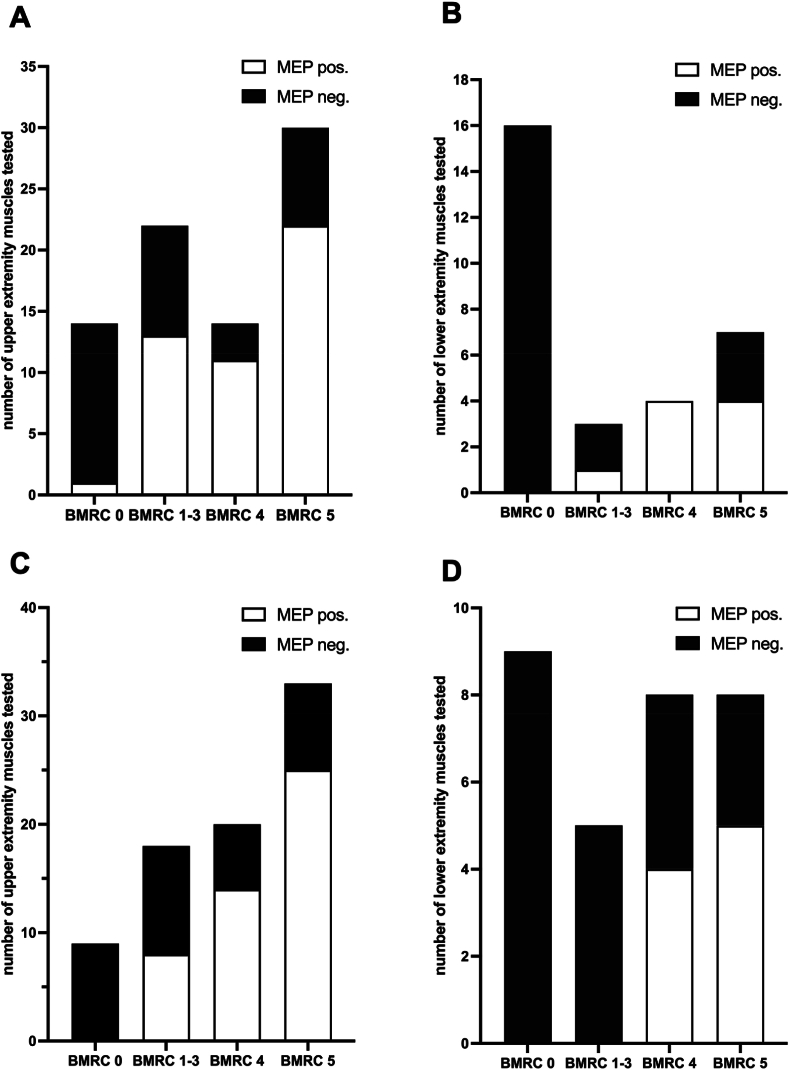
Correlation between muscle strength and nTMS motor status.

In total, recordings of nTMS and IONM were available for 88 muscles, with 62 from the UE and 26 from the LE. Intraoperatively, positive MEPs derived from IONM (MEPs_IONM_) were observed for 38 (61.3%) of 62 UE muscles, and in nTMS, positive MEPs_TMS_ were observed for 41 muscles (66.1%) (p = 0.581) (Fig. 2). For LE, 5 of 26 muscles (19.2%) were MEP-positive in IONM and 7 of 26 muscles (26.9%) were MEP-positive for nTMS (p = 0.625) (Fig. 2). In 6 cases (6.8%) (5 UE and 1 LE), nTMS did not evoke MEPs_TMS_, which were true positive MEPs_IONM_ during IONM. In 11 cases (12.5%) (8 UE and 3 LE), IONM did not evoke MEPs_IONM_, which were true positive MEPs_TMS_ during the nTMS examination.

Additionally, the findings of positive MEPs from IONM and nTMS were combined. For clinical motor activity in the corresponding UE muscle, a positive predictive value of 97.9% was achieved, with a sensitivity of 85.2% and a specificity of 88.9% (Table 4). For LE, a positive predictive value of 100% was achieved, with a sensitivity of 57.1% and a specificity of 100% (Table 4). A correlation between MEPs_IONM_ and MEPs_TMS_ was additionally performed for a clinical motor activity BMRC ≥4 (Table 4).

### 3.6 Data availability statement

In the interest of patient privacy, all the raw data collected from individual cases is imparticipable. The anonymous datasets used and analyzed during the current study, the study protocol, and the statistical analysis plan are available upon reasonable request from the corresponding author to researchers who provide a methodologically sound proposal. Proposals should be directed to maximilian.schwendner@med.uni-heidelberg.de; to gain access, data requestors will need to sign a data access agreement.

## 4 Discussion

The aim of this study was to analyze the diagnostic and prognostic value of nTMS in SCI patients. Comparing nTMS and IONM, a higher rate of muscles showing motor function at follow-up was observed for nTMS. The overall highest sensitivity for motor function at follow-up was observed for combining nTMS and IONM.

Regarding the structured clinical examination, the optimal timing of the neurological examination to predict neurological prognosis is a topic of ongoing discussion. While daily clinical examinations are essential in SCI patients, current literature suggests that neurological assessments for outcome prediction should be conducted between 24 h and 1 week post-injury, as an examination during this period is the most reliable and predictive of neurological outcome (van Middendorp et al., 2009; Marino et al., 2011; El Tecle et al., 2018). Regarding long-term clinical outcomes, the initial neurological status at presentation also plays a crucial role, which requires an accurate clinical examination. The majority of motor recovery and ASIA grade improvement occurs within the first 6–9 months, with the most rapid rate of motor recovery occurring in the first 3 months after injury (Kirshblum et al., 2021; Marino et al., 1999). The total motor recovery is greater for patients with initial ASIA B than ASIA A, and greater after initial ASIA C than with ASIA A or B (Marino et al., 1999, 2011; Kirshblum et al., 2021).

In terms of imaging and clinical outcome prediction, magnetic resonance imaging (MRI) studies in patients suffering from acute SCI have indicated a correlation between the T2-positive edema signal and injury severity in the clinical examination (Parashari et al., 2011; Le et al., 2015). Parashari et al. classified patients based on the presence of a sizable focus of hemorrhage in the spinal cord and the extent of spinal cord injury, categorizing injuries as either less than or greater than 3 cm on MRI (Parashari et al., 2011). Talbott et al. developed the Brain and Spinal Injury Center (BASIC) score, a reproducible method for assessing the severity of acute cervical spinal cord injury (Talbott et al., 2015). It defines five distinct patterns of intramedullary spinal cord T2 signal abnormality, observed in the axial plane at the injury epicenter and strongly correlates with neurological symptoms at the time of both hospital admission and discharge (Talbott et al., 2015).

For additional diagnostic tools for patients with SCI, intraoperative transcranial electric stimulation using MEPs is frequently applied. This method not only enables real-time monitoring of the motor system's integrity but also offers prognostic information. Dhall et al. demonstrated in a cohort of 32 patients suffering from acute traumatic spinal cord injury that the presence of MEPs in ION was a significant predictor of ASIA at discharge (Dhall et al., 2018). The group of severe SCI patients with elicitable MEPs showed a superior improvement in ASIA grade compared to patients who lacked any MEPs in IONM (Dhall et al., 2018).

In the majority of patients in this study, transcranial electric stimulation was performed intraoperatively and MEPs_IONM_ were recorded. In addition, nTMS motor mapping was successfully performed 4.5 ± 4.1 (1–14) days after trauma in all patients. It was feasible to perform nTMS in the intensive care unit (ICU), and due to its non-invasive nature, the procedure was well tolerated by the patients. When performing nTMS in these patients, we observed increased MEP latencies overall. Similar findings were observed in nTMS studies in patients with myelopathy (Nakamae et al., 2010). Furthermore, a higher rMT was observed in the patients in this study compared to healthy volunteers, indicating an impaired excitability of the motor system in general.

Comparing IONM and nTMS, both methods stimulate the primary motor cortex; MEPs_IONM_ are frequently recorded using needle electrodes, while MEPs_TMS_ are derived from surface electrodes. nTMS offers precise cortical stimulation with an accuracy of a few millimeters, whereas the stimulation site for transcranial electric stimulation is anatomically predefined and does not provide focal stimulation. However, transcranial electric stimulation allows for higher stimulation intensities compared to nTMS because the patient is under general anesthesia. Regarding the clinical findings, IONM showed positive MEPs_IONM_ in 6 muscle recordings that nTMS did not show. The potential causes might be the technical specifications to apply higher stimulation intensities when using transcranial electric stimulation and a higher spatial resolution and sensitivity of MEPs when using needle electrodes. On the other hand, we observed cases with MEPs_TMS_ during nTMS which were not registered during IONM. These findings might be related to the effects of intraoperative anesthesia, surgery or early neurological recovery, comparable to an early postoperative neurological recovery observed in the clinical examination.

Over time, the positive predictive value increased from the initial nTMS examination to the follow-up, accompanied by a reduced number of false negatives (Table 4). This correlates with higher rates of muscles showing motor function at follow-up (FU) compared to the clinical examination at the nTMS examination (Table 3). At follow-up, we also observed higher rates of muscles with clinical motor function (BMRC ≥1) despite the absence of MEPs compared to the status at the nTMS examination. This change led to a lower negative predictive value for the absence of clinical motor function in muscles without MEPs (Table 4). Both methods, IONM and nTMS, demonstrate high positive predictive values in this study, which allow positive results on IONM and especially nTMS to predict motor function at follow-up. The highest sensitivity for detecting motor function in muscles below the level of injury was achieved when combining positive results from IONM and nTMS postoperatively (Table 4). In this study, a ratio of positive MEPs from IONM and nTMS combined ≥50.0% indicated ASIA D at follow-up for UE SCI. In patients with ASIA D at FU with SCI only affecting LE motor function, the ratio of positive MEPs from IONM and nTMS combined was 100% and for nTMS alone was 50% (Table 5).

Although lower extremity MEPs were less sensitive, upper extremity MEPs were highly sensitive. Thus, TMS confers a high negative predictive value for cervical spinal cord injuries, which would be expected to affect upper extremity function.

As one performs and interprets the TMS, it is useful to keep in mind the anatomic level of injury (cervical vs thoracic). For cervical cases, the presence of UE MEPs_TMS_ is reassuring, even when LE MEPs_TMS_ cannot be elicited. Conversely, in thoracic injuries, UE MEPs_TMS_ may be used as a positive control, and the absence of LE MEPs is not as reliable a predictor of outcome. Further studies are needed to enhance the sensitivity of MEPs_TMS_ in LE.

Including different stimulation techniques, such as paired-pulse stimulation, and utilizing coils with varied architectures, such as H-coils, may improve the sensitivity of LE TMS results and should be addressed in further studies (Sollmann et al., 2020).

### 4.1 Multi-modal prognosis in spinal cord injury

While the clinical evaluation of motor strength remains the gold standard in assessing patients with SCI, it is inherently subjective, relying on the patient's active participation and the examiner's judgment. In contrast, MEPs obtained through nTMS provide a quantifiable and objective assessment. nTMS is a non-invasive technique that can be performed on both conscious and comatose patients, making it a valuable diagnostic tool in the management of SCI. Optimal timing for nTMS evaluation appears to be two to seven days post-injury. Although nTMS findings should ideally be interpreted in conjunction with IONM data, they can also be valuable as a standalone assessment.

### 4.2 Limitations

Firstly, this study was conducted as a single-center pilot study, which may limit the generalizability of the findings. The sample size was relatively small, with the number of subjects limited to 20 overall. This small sample size reduces the statistical power of the study and may affect the robustness of the results. Additionally, the timing of the nTMS examination varied between individual patients, and it was not performed within 7 days in 3 patients (15%). This variability could potentially influence the outcomes as well as the calculated PPV and NPV.

Moreover, in the clinical setup, the nTMS examination was restricted to MEPs_TMS_ of BCS, APB, and TA for all subjects. A broader range of muscles matching spinal levels according to the ASIA could provide a more comprehensive understanding of the patient's neuromuscular status.

Regarding the clinical findings in this study, a relatively low sensitivity for LE muscle motor outcomes was observed, which has to be taken into account when interpreting these results.

Future studies should consider a multicenter design with a larger sample size and standardized timepoints of assessment, as well as a wider array of muscles and parameters for nTMS examinations, to enhance the reliability and applicability of the findings.

## 5 Conclusion

nTMS serves as a tool to provide an objective assessment of the integrity of the motor system for both the upper and lower extremities in patients with acute SCI. Positive MEPs, whether elicited via TMS or IOMN, below the level of injury are a strong indicator of a favorable long-term motor outcome. Especially in combination with IONM results, nTMS motor mapping can be used as an adjunct to objectively evaluate the motor status and predict long-term neurological outcome after acute traumatic spinal cord injury.

### 5.1 Transparency, Rigor and reproducibility summary

This study was not formally registered because of the complete absence of invasiveness and the non-randomized study design. The analysis plan was not formally registered, but the member with primary responsibility for the analysis (lead author) certifies that the analysis plan was pre-specified. A sample size of 20 subjects was planned based on the availability of patients matching the inclusion criteria and a limited time span for data collection. In total, primary measurements were collected and analyzed from 20 subjects included in this study. Data analyses were performed by investigators who were aware of relevant characteristics of the participants including the clinical status and measurements derived from intraoperative neuromonitoring and transcranial magnetic stimulation. Data were acquired between January 2022 and July 2024. The key inclusion criteria and outcome evaluations (ASIA classification) are established standards. In the interest of patient privacy, all the collected raw data of individual cases is imparticipable. This paper will be published under a Creative Commons Open Access license, and upon publication will be freely available at https://www.liebertpub.com/loi/neu.

## Submission statement

This manuscript is original and has not been submitted in part or whole elsewhere.

## Previous presentations

Parts of this work have been presented as an oral presentation at the NBS Symposium 2024 on October 12th in Berlin, Germany.

## Authorship contribution statement

Conceptualization: Maximilian Schwendner, Phiroz E. Tarapore; Methodology: Maximilian Schwendner, Anthony M. DiGiorgio, Michael C. Huang, Geoffrey T. Manley, Phiroz E. Tarapore; Formal analysis and investigation: Maximilian Schwendner, Marianne Kanaris, Phiroz E. Tarapore; Writing - original draft preparation: Maximilian Schwendner, Phiroz E. Tarapore; Writing - review and editing: Maximilian Schwendner, Marianne Kanaris, Anthony M. DiGiorgio, Michael C. Huang, Geoffrey T. Manley, Phiroz E. Tarapore; Funding acquisition: Phiroz E. Tarapore; Resources: Phiroz E. Tarapore; Supervision: Phiroz E. Tarapore; All authors agreed on the publication of the final version of this manuscript.

## Authors’s disclosures

All authors have completed the ICMJE uniform disclosure form at www.icmje.org/coi_disclosure.pdf and declare no support from any organization for the submitted work. MS is a consultant for Level Ex Inc (Chicago, Illinois, United States) and Sonovum GmbH (Leipzig, Germany). AD receives grant funding from 10.13039/100008127DePuy Synthes, The 10.13039/100019004Mercatus Center at George Mason University and the 10.13039/100013861Charles Koch Foundation.

## Funding

MS is funded by the clinician-scientist program of Heidelberg University, Faculty of Medicine. Otherwise, this study did not receive any other specific grant from funding agencies in the public, commercial, or not-for-profit sectors. This trial was funded mainly by institutional grants from the Department of Neurosurgery, Zuckerberg San Francisco General Hospital 10.13039/100008069University of California San Francisco.

## Appendix A Supplementary data

Fig. 1 presents the case of a 68-year-old patient with cervical spinal cord injury classified as American Spinal Injury Association Impairment Scale C, following a scooter accident. Sagittal magnetic resonance imaging before (A) and after (B) dorsal instrumentation and decompression of the levels C2-T1 is displayed. Navigated transcranial magnetic stimulation (nTMS) was conducted (C, D). Computed tomography imaging was used for navigating the magnetic stimulation coil (C). EMG recording was performed for the biceps brachii muscle (green), abductor pollicis brevis muscle (yellow), and tibialis anterior muscle (blue) (D).

Fig. 2 illustrates muscle strength according to the British Medical Research Council Scale (BMRC) and motor-evoked potentials (MEPs) for upper extremity (UE) and lower extremity (LE). Analysis was performed for examinations at the time of the navigated transcranial magnetic stimulation (nTMS) (A + B) and at follow-up (C + D). For UE, the biceps brachii muscle and abductor pollicis brevis muscle were examined. For LE, the tibialis anterior muscle was recorded.
